# Component Composition and Biological Activity of Various Extracts of *Onosma gmelinii* (Boraginaceae)

**DOI:** 10.1155/2022/4427804

**Published:** 2022-07-22

**Authors:** Sergey V. Shilov, Gulbaram O. Ustenova, Lashyn N. Kiyekbayeva, Ilya S. Korotetskiy, Natalia V. Kudashkina, Natalya V. Zubenko, Raikhan A. Parenova, Ardak B. Jumagaziyeva, Zhanar A. Iskakbayeva, Sabina T. Kenesheva

**Affiliations:** ^1^Department of Pharmaceutical Technology, Asfendiyarov Kazakh National Medical University, Almaty 050000, Kazakhstan; ^2^Virology Laboratory, JSC Scientific Center for Anti-Infectious Drugs, Almaty 050060, Kazakhstan; ^3^Kazakh-Russian Medical University, Faculty of Pharmacy, Public Health and Nursing, Almaty 050000, Kazakhstan; ^4^Department of Pharmacognosy with a Course in Botany and the Basics of Phytotherapy, Faculty of Pharmacy, Bashkortostan State Medical University, Ufa 450008, Russia; ^5^Department of Engineering Disciplines, Asfendiyarov Kazakh National Medical University, Almaty 050000, Kazakhstan; ^6^Microbiology Laboratory, JSC Scientific Center for Anti-Infectious Drugs, Almaty 050060, Kazakhstan

## Abstract

*Onosma* roots are widely used in traditional medicine to treat various diseases throughout the world. In this study, for the first time, we investigated the component composition and biological activity of various extracts from the roots of *Onosma gmelinii* collected in the highlands of the Kakpakty Mountains of the Almaty region (Republic of Kazakhstan). Extracts were obtained by three different methods: percolation extraction, ultrasound-assisted extraction, and supercritical carbon dioxide extraction. The component composition of the extracts was determined by gas chromatography/mass spectrometry (GC/MS), naphthoquinones by thin-layer chromatography (TLC), and spectrophotometric method. In this study, the presence of shikonin and its derivatives in the extracts was confirmed. The concentration of naphthoquinones during CO_2_ extraction was about 40%, during ultrasonic extraction about 3%, and during percolation extraction about 1.3%. The GC-MS method identified 69 chemical compounds in the ultrasonic extract, 46 compounds in the CO_2_ extract, and 51 compounds in the percolation extract. The extracts were tested on a panel of bacteria and viruses: two Gram-negative bacteria (*Escherichia coli* ATCC 8739, *Pseudomonas aeruginosa* ATCC 9027); nine Gram-positive bacteria (*Staphylococcus aureus* ATCC 6538-P, *Staphylococcus aureus* ATCC BAA-39, *Staphylococcus epidermidis* ATCC 51625, *Staphylococcus epidermidis* ATCC 12228, *Streptococcus pyogenes* ATCC 19615, *Streptococcus pneumoniae* ATCC BAA-660, *Enterococcus hirae* ATCC 10541, *Enterococcus faecalis* ATCC 51575, *Enterococcus faecium* ATCC 700221); and two fungal species (*Candida albicans* ATCC 10231, *Candida albicans* ATCC 2091); five subtypes of influenza virus A (A/FPV/Weybridge/78 (H7N7), A/Swine/Iowa/15/30 (H1N1), A/black-headed gull/Atyrau/743/04 (H13N6), A/FPV/Rostock/1934 (H7N1), A/Almaty/8/98 (H3N2)). The root extracts of *Onosma gmelinii* showed antibacterial activity in different degrees against all tested Gram-positive bacterial strains, while no inhibitory effect on Gram-negative bacteria was observed. The results indicated that the ultrasonic extract effectively inhibits the growth of the majority of tested Gram-positive bacteria (MBC from 18.3 to 293.0 *µ*g/mL). CO_2_ extract had the greatest bactericidal activity (MBC from 0.1 to 24.4 *µ*g/mL). Percolation extract insignificantly inhibited bacterial growth (MBC from 2343.8 to 4687.5 *µ*g/mL). CO_2_ extract and ultrasonic extract significantly reduced the activity of *C. albicans.* The results of the antiviral action showed that the ultrasonic extract has the greatest effectiveness against different subtypes of the influenza virus A, while other extracts did not show significant activity.

## 1. Introduction

Plants are an inexhaustible source of biologically active substances, based on which medicines are created. Since ancient times, people have used traditional herbal medicine to treat many diseases [1, 2]. It is believed that the use of drugs of natural origin together with synthetic ones makes it possible to reduce or completely neutralize the side effects of the latter. Widespread use of herbal preparations is observed not only in Asian countries but also in developed countries of Europe and North America [3]. Traditional medicine, which mainly uses combinations of herbs prescribed as medicines, plays a special role in China, where it accounts for approximately 40% of the Chinese pharmaceutical market [4]. According to WHO, almost 80% of the world's population within the organization of first aid uses mainly herbal preparations [3]. To date, out of 320 thousand described plant species, about 21 thousand species are used in traditional medicine. The register of medicinal plants is constantly updated. Most of the medicinal plants are recognized by official medicine and are widely used in the production of pharmaceuticals. Phytopreparations are well combined with other methods of treatment, their use allows not only for curing some diseases but also for preventing their further development. It has been established that more than 40% of drugs on the pharmaceutical market contain medicinal plant raw materials in their composition.

The genus *Onosma* L. belongs to the family Boraginaceae Juss., subfamily Boraginoideae, is included in the tribe Lithospermeae. The genus *Onosma* is represented by perennial herbaceous plants, subshrubs, and prostrate subshrubs. *Onosma* species have been used in traditional medicine for centuries for the treatment of various diseases throughout the world [5, 6]. The genus *Onosma* L. (Boraginaceae) represents about 150 known species distributed mainly in the dry regions of the Mediterranean and territories from Western and Central Asia to Western China. However recent systematic botanical studies have increased the number of species in this genus to over 230 species [5, 7].

Some types of *Onosma* are part of multicomponent herbal preparations. The Indian drug “Cystone,” containing an extract of *Onosma bracteatum* stems, is used for urolithiasis, reduces the content of stone-forming substances, has an anti-inflammatory, antimicrobial, antiseptic, and diuretic effect, and reduces irritation of the bladder mucosa [8]. *Arnebia euchroma, Arnebia guttata, Lithospermum erythrorhizon, Onosma paniculatum, Onosma exsertum, Onosma confertum, Onosma hookerii,* and *Onosma waltonii* are used as interchangeable sourcing plants for the Chinese Materia Medica known as “Zicao.” The Zicao is used externally for the treatment of burns, frostbite, eczema, ulcers, and inflammation and as an antiparasitic, antipyretic, and antiseptic [9]. *Onosma* roots are widely used in the treatment of infectious diseases such as hemorrhoids, tonsillitis, and bronchitis and have an important potential in the recovery of wounds and burns [10]. Some *Onosma* species are effective against abdominal pain, eye diseases, ear diseases, lung diseases, blood diseases, urinary calculi, dysuria, piles, fever, itching, and thirst [6, 11]. Flowers of these species can also be used in the treatment of rheumatism and are also used as a cardiac stimulant agent [12]. Infusions of herb *Onosma* have hypotensive, sedative, diuretic, myotropic, and antipyretic effects, reduce capillary fragility and vascular permeability, and lower blood pressure [13]. *Onosma* root extracts have antioxidant, anthelmintic, antifungal, and antimicrobial activity [14, 15] and also have an antitumor effect [16]. Most members of the *Onosma* are known to produce shikonin derivatives. Particularly the red dye of *Onosma* sp. has been used since ancient times in the textile and food industries [17]. Moreover, since this pigment is a natural dye, it is often used for medical and cosmetic applications [6, 18].

Shikonin and its derivatives exhibit activity against HIV type 1, possess anti-ADV3 (adenoviral type 3) properties, and are able to inhibit HCV replication [19]. The activity of naphthoquinones against influenza A is poorly studied. Influenza A viruses with a high degree of genome variability are etiological agents of dangerous infectious diseases in humans and animals that can occur in the form of extensive epizootics, epidemics, and pandemics with high mortality. At the same time, some of the newly emerging strains show resistance to existing anti-influenza drugs. Therefore, the problem of developing and searching for new drugs to protect against influenza infection, including both prophylactic and therapeutic drugs, seems extremely important and especially relevant.

In Kazakhstan, three species of the *Onosma* genus have gained the greatest popularity in traditional medicine: *Onosma dichroantha* contains saponins and alkaloids, flowers are used in the treatment of impotence, and root juice is used as an analgesic for otitis media. *Onosma simplicissima* helps lower blood pressure and increase the amplitude of heart contractions and has an antipyretic effect. *Onosma transrhymnensis* is used for headaches and as a sedative, hypotensive, diuretic, and myotropic agent [13].

Our research area includes the little-studied wild plant *Onosma gmelinii*. It is a perennial herbaceous plant 20–50 cm high. The root is thin, dark purple, or brownish, usually with 1–5 flowering stems and a rosette of leaves at their base, stems are bluish, rarely green, unbranched, ascending, densely covered with protruding thin long bristles, which are seated with short stiff protruding hairs. Leaves are also covered with long protruding bristles and stiff hairs. Basal leaves are long petiolate, oblanceolate; stem leaves are upward-directed, lanceolate, sessile, gradually narrowed towards the base, obtuse, or shortly pointed, with inflorescences terminal, unbranched, with numerous crowded flowers. Corolla is light yellow, later turning brown, tubular-campanulate, gradually expanded upward; throat is obscurely pubescent outside, glabrous inside; lobes are broadly triangular. Nutlets are grey-brown, up to 5 mm long, lustrous, smooth, gradually tapering to beak, not gibbous. It blooms in May–July and bears fruit in July–August [20]. It grows mainly on rocky and dry gravelly slopes, rocks, dry terraces in the lower and middle mountain belts, rarely in the steppes and mountain steppe belt. Spread area is Eastern Siberia, Kazakhstan, Kyrgyzstan, Tajikistan, Uzbekistan, Turkmenistan, Mongolia, China, and Kashmir.

It belongs to technical plants, the roots of which have been a source of red dye for colouring wool and oils for many years [21], but the chemical composition of the plant is insufficiently studied still.

This study aims to explore the component composition of various extracts from the roots of *Onosma gmelinii* obtained by percolation extraction, ultrasound-assisted extraction, and supercritical carbon dioxide extraction. For the first time, their antimicrobial activity against pathogenic microorganisms and antiviral activity against various strains of the influenza virus was established. The results will allow us to select the most effective and cost-effective extract of the roots of *Onosma gmelinii* to develop antibacterial drugs for medical practice.

## 2. Material and Methods

### 2.1. Plant Material

Roots of *Onosma gmelinii* were collected in the flowering phase in August, 2019, in the highlands of the Kakpakty Mountains (Kokbel village, Raiymbek district of Almaty region, Kazakhstan). Sample of plant was identified by a Botanist at the Department of Biodiversity and Bioresources, Faculty of Biology and Biotechnology, Al-Farabi Kazakh National University, Almaty, Kazakhstan.

The samples were washed thoroughly in running tap water to remove soil particles and adhered debris and finally washed with sterile distilled water. The roots of plant were dried in a well-ventilated room at a temperature of 25 ± 5°C. The dried roots were finely powdered using an electric grinder to a fraction of 0.5–2.0 mm and stored in airtight containers until use.

### 2.2. Preparation of Plant Extracts

The extraction of plant raw materials was performed in three different methods.

#### 2.2.1. Percolation Extraction (P)

40 g of powdered plant material was packed in a percolator, with adding 80 mL of 70% ethanol, and macerated overnight [22]. The percolation process was carried out at a moderate rate until the plant raw materials were completely depleted. Liquid extracts obtained were filtered through ashless filter paper and concentrated using a rotary evaporator (IKA RV 05 basic, Germany). The yield of the dry extract was 7.56 g (18.9%).

#### 2.2.2. Ultrasound-Assisted Extraction (UAE)

40 g of powdered plant material was soaked in 800 mL of 70% ethanol for 30 minutes [22]. Then the mixture was subjected to a double ultrasonic extraction in an ultrasonic bath (Elmasonic S90H, Germany, 400 W, 37 kHz) for 30 minutes each. Water in the ultrasonic bath was circulated at a temperature of 40 ± 1°C. The supernatant was similarly processed as described in percolation to get dried UAE extract of *Onosma gmelinii*. The yield of the dry extract was 4.47 g (11.2%).

#### 2.2.3. Supercritical Carbon Dioxide Extraction (CO2-SE)

CO_2_ extraction of *Onosma gmelinii* roots was carried out in a supercritical fluid extractor (Thar, USA) [22]. 500 g of powdered plant material was placed into stainless steel extraction cell. The cosolvent pump was filled with 90% ethanol. The temperature was 45–50°C, while the pressure used was above 100 bar. The total extraction time was 70 min, consisting of 10 min of static extraction followed by 60 min of dynamic extraction. The extract was collected in a glass vessel cooled by CO_2_ expansion and then concentrated by the rotary evaporator (IKA RV 05 basic, Germany). The yield of the thick extract was 12.44 g (2.5%).

### 2.3. Determination of Naphthoquinones

500 mg (exact weight) of the test extract was completely dissolved in 100 mL of chloroform. The qualitative determination of naphthoquinones in the test solutions was carried out by thin-layer chromatography (TLC) [23, 24]. A 20 *µ*L of test samples of different extracts was applied to the TLC plate (aluminum foil-backed TLC plate coated with silica gel 60 F254, Merck, 0.2 mm layer thickness) separately [25]. It was positioned 10 mm from the bottom of the plate, and then the plate was developed to a distance of about 10.5 cm using hexane-acetone (20 : 1) as the mobile phase. The plates were then removed and air-dried for 15 minutes. The naphthoquinones formed red zones, the colours of which changed to violet on treatment with ammonia vapour. Detection of the phytochemicals was conducted by comparison of Rf values and spot colours with literature data.

The quantitative determination of the amount of naphthoquinones in terms of shikonin in the extracts was carried out by the spectrophotometric method. Under the condition of the high content of the number of naphthoquinones in the test extract, the test solution was diluted with chloroform until an optical density of 0.2 to 0.8 was reached; the dilution coefficient was used in the calculations. The optical density of the test solutions was measured on a spectrophotometer at a wavelength of 525 nm. Calculation of the amount of content naphthoquinones was performed based on shikonin by specific absorption rate shikonin (*A*_1*см*_^1%^=430) [26].

### 2.4. Component Composition Determination

#### 2.4.1. GC-MS Analysis

The chemical composition of extracts was determined on a gas chromatograph with an Agilent 6890°N/5973°N mass spectrometric detector. Chromatography conditions were as follows: sample volume 1.0 *μ*L and sample inlet temperature 250°C, without dividing the flow. The separation was carried out using a DB-WAXETR chromatography capillary column with a length of 30 m, an inner diameter of 0.25 mm, and a film thickness of 0.25 *μ*m at a constant carrier gas (helium) velocity of 1 mL/min. The chromatographic temperature was programmed from 40°C (holding 0 min) to 240°C with a heating rate of 5°C/min (holding 10 min). The detection was carried out in the SCAN m/z 34–750 mode. Agilent MSD ChemStation (v.1701EA) software was used to control the gas chromatography system, recording, and processing of the results and data.

#### 2.4.2. Identification of Compounds

Interpretation on mass spectrum GC-MS was conducted using the databases of National Institute Standard and Technology (NIST'02) and Wiley 7th edition. The percentage of components was calculated automatically based on the peak areas of the total ion chromatogram. The components were identified by mass spectra and retention times.

### 2.5. Antiviral Activity Determination

#### 2.5.1. Viruses and Cells

Madin-Darby canine kidney (MDCK) cell lines were obtained from RSE “Scientific Research Institute for Biological Safety Problems” CS MES RK. The cell line was grown in Dulbecco's Modified Eagle Medium (DMEM; Sigma-Aldrich) supplemented with 10% fetal bovine serum (FBS), penicillin (100 units/mL), streptomycin (100 *μ*g/mL), and amphotericin B (0.25 *μ*g/mL) at 37°C and 5% CO_2_ atmosphere.

Influenza viruses A/FPV/Weybridge/78 (H7N7), A/Swine/Iowa/15/30 (H1N1), A/black-headed gull/Atyrau/743/04 (H13N6), A/FPV/Rostock/1934 (H7N1), and А/Almaty/8/98 (H3N2) were obtained from RSE «Institute of Microbiology and Virology» SC MES RK. Influenza virus was propagated in MDCK cells at 37°C for 72 h, then virus-containing fluid was harvested and centrifuged to remove the cell debris at 300 g for 15 min, and clarified supernatant was stored at −70°C [27]. The 50% tissue culture infective dose (TCID_50_) was determined using Reed and Muench [28].

#### 2.5.2. Test Extracts

The extracts performed by percolation and ultrasonic extraction were dissolved in DMSO, and supercritical carbon dioxide extract was dissolved in 96% ethanol. The choice of solvent was determined by the physicochemical properties of the extracts.

The final maximal DMSO or 96% ethanol concentration in the assay wells with the highest sample input (1%) was well tolerated by the cells. The cytotoxicity and antiviral activity were determined by treating the monolayers with twofold serial dilutions of test extracts.

#### 2.5.3. Cytotoxicity Assay

The cytotoxicity of plant extracts was determined by the MTT method [29, 30]. The absorbance of each well was measured at the wavelength of the main filter of 540 nm and a reference wave of 620 nm in a microplate reader (Tecan Sunrise RC.4, Austria). The median cytotoxic concentration (CC_50_) was calculated as the concentration of the plant extracts that reduced the viable cells to 50% of the untreated control [31, 32].

#### 2.5.4. Antiviral Assay

*In vitro* antiviral activity against influenza viruses was determined according to two schemes: therapeutic and virus inhibition [31]. All studies were tested in four replicates. For both schemes, IC_50_ (half maximal inhibitory concentration) and selective index (SI) were calculated. The SI was calculated as the ratio of CC_50_/IC_50_ [31, 33].

*(1) Therapeutic Activity*. A therapeutic activity assay was performed to evaluate the inhibitory effects of the investigated compounds on viral cycle's intracellular stage, such as replication, particle assembly, or budding. Briefly, confluent MDCK cells were infected with the influenza virus at a dose of 100 TCID_50_/0.2 mL and incubated for 1 hour at 37°C. Then, the contents of the wells were removed, and the final dilutions of the test extract were added. Serum-free DMEM was used as negative control and an untreated virus as a positive control. The plates were incubated for 72 h in a CO_2_ incubator at 37°C in 5% CO_2_. The therapeutic activity of the test compound against the influenza virus was detected as a result of a decrease in the titer of the residual virus infectivity in the hemagglutination reaction [27].

*(2) Virus Inhibition Activity*. A viral inhibition assay was performed to determine the direct effect of an extract on viral particles. Briefly, the serially diluted compound was preincubated with the virus at 100 TCID_50_/0.2 mL at 37°C for 1 h. Then serial 10-fold dilutions of virus-extract mixture were prepared. The growth medium was removed from the wells of the cell culture plate, and the prepared dilutions were added starting from the initial one. Serum-free DMEM was used as negative control and an untreated virus as a positive control. The plate was incubated for 72 h at 37°C in 5% CO_2_. The results were evaluated based on the presence or absence of a virus in the cell culture in the hemagglutination test [27]. The titer of the residual virus infectivity was calculated by Reed and Muench [28].

### 2.6. Antimicrobial Activity Determination

#### 2.6.1. Microbial Strains

The antibacterial activity was evaluated using a panel of pathogenic strains including Gram-negative bacteria: *Escherichia coli* ATCC 8739, *Pseudomonas aeruginosa* ATCC 9027; Gram-positive bacteria: *Staphylococcus aureus* ATCC 6538-P, *Staphylococcus aureus* ATCC BAA-39 (multidrug resistant, MRSA), *Staphylococcus epidermidis* ATCC 51625 (methicillin resistant), *Staphylococcus epidermidis* ATCC 12228 (VRSA), *Streptococcus pyogenes* ATCC 19615, *Streptococcus pneumoniae* ATCC BAA-660 (multidrug resistant), *Enterococcus hirae* ATCC 10541, *Enterococcus faecalis* ATCC 51575 (gentamicin, streptomycin, and vancomycin resistant), and *Enterococcus faecium* ATCC 700221 (vancomycin and teicoplanin resistant); and two fungal strains: *Candida albicans* ATCC 10231, *Candida albicans* ATCC 2091.

The storage, maintenance, and preparation of working culture suspensions were carried out following established procedures [34].

#### 2.6.2. Antimicrobial Assay

A 48-well plate was used to determine the antimicrobial activity [35, 36]. A twofold serial dilution series of the test extract using Mueller Hinton broth (Mueller Hinton broth (M391), HiMedia, India) for bacterial testing and Sabouraud broth (Fluid Sabouraud medium (M013), HiMedia, India) for fungal testing was prepared in a separate 48-well microplate. The initial concentration for CO_2_ extract was 100000.0 *μ*g/mL, and for ultrasound-assisted and percolation extraction it was 150000.0 *μ*g/mL. Antibiotics ampicillin (the initial concentration was 4000.0 *μ*g/mL) and nystatin (the initial concentration was 11100.0 *μ*g/mL) were used as reference drugs for bacteria and fungi, respectively. The medium and test strain was used as a positive control to confirm growth for each test strain. For each test strain, a suitable nutrient broth (Mueller Hinton broth for bacterial testing or Sabouraud broth for fungal testing) without test material was used as a negative control.

To all wells with dilution and positive control, 50.0 *μ*L of the tested strain at a concentration of 1.5 × 10^6^ CFU/mL was introduced. All samples were incubated at 37 ± 1°C for 24 h. After incubation, the samples were seeded on Petri dishes with an appropriate dense medium to determine the live strains and incubated at 37 ± 1°C for 24 hours (for bacteria) and at 22 ± 1°C for 48 hours (for fungi). The results were detected visually by the presence/absence of visible growth of microorganisms on the surface of the dense nutrient medium. The minimum bactericidal/fungicidal concentration (MBC/MFC) was considered the lowest concentration that suppressed microorganisms growth.

### 2.7. Statistical Analysis

The statistical evaluation of the data was done using one-way analysis (ANOVA) using GraphPad Prism 6 application software package, version 6.01 for Windows. The significance values were set at *p* < 0.05.

## 3. Results and Discussion

### 3.1. Component Composition of Extracts

#### 3.1.1. GC-MS of Extracts

**Figure 1:**
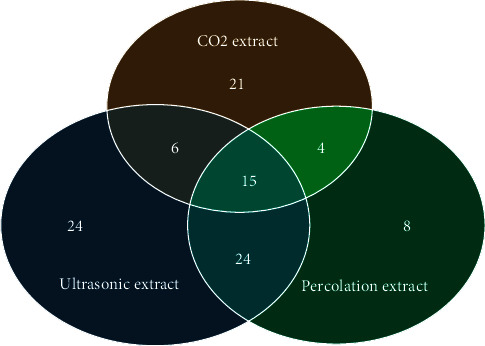
Venn diagram of the distribution of phytochemical components.

The extracts were rich phytochemical components in different concentrations. A total of 102 chemical compounds were detected (Table 1 and Figure 1).

The GC-MS screening has revealed that ultrasound-assisted extraction is the most optimal method of extracting biologically active substances from the roots of *Onosma gmelinii*. 69 components were identified in the ultrasonic extract, 46 in the CO_2_ extract, and 51 in the extract performed by percolation. Flavonoids are such as 2,3-dihydro-3,5-dihydroxy-6-methyl-4h-piran-4-one (3.51%) with anti-inflammatory, analgesic, and antimicrobial activity [37, 38]; organic compounds such as 5-hydroxymethylfurfural (10.67%), exhibiting antioxidant and antiproliferative properties [38], were found among the main components of the ultrasonic extract.

Only 15 chemical components were found in all three extracts: monocarboxylic acids and its derivatives such as acetic acid, 3-methyl-2-butenoic acid, 3-methyl-3-butenoic acid, 2-methyl-2-butenoic acid, 3-hydroxy-3-methyl-butanoic acid; triterpenes such as squalene; fatty acids and fatty acid derivatives such as propanoic acid, 2-methyl-propanoic acid, oleic acid, hexadecanoic acid, ethyl ester hexadecanoic acid, ethyl ester 9,12-octadecadienoic acid, 9,12-octadecadienoic acid, and 9,12,15-octadecatrienoic acid were found among the main classes of compounds.

However, GC-MS did not allow determining the presence of naphthoquinone pigments in the extracts, which are well-known components of plants of the *Boraginaceae* family. Therefore, thin-layer chromatography (TLC) and spectrophotometric method were used to detect naphthoquinones.

#### 3.1.2. Determination of Naphthoquinones

**Figure 2:**
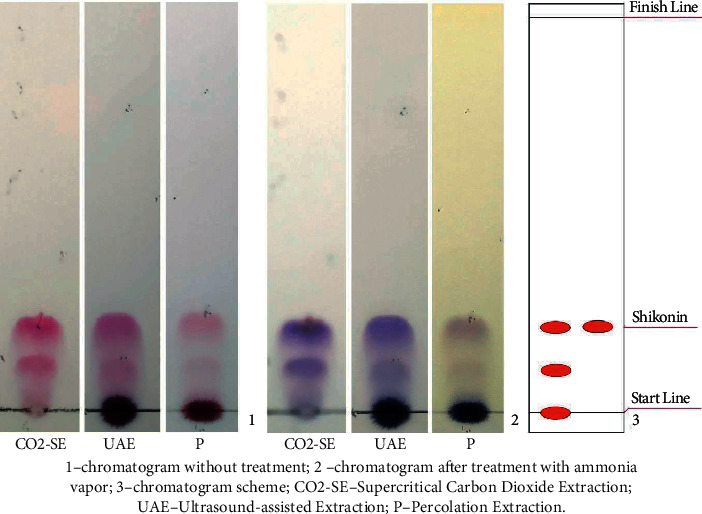
Thin-layer chromatogram of different extracts from the roots of *O. gmelinii*. (1) chromatogram without treatment; (2) chromatogram after treatment with ammonia vapour; (3) chromatogram scheme; CO2-SE: supercritical carbon dioxide extraction; UAE: ultrasound-assisted extraction; P: percolation extraction.

Unlike other chromatographic techniques, TLC is a simple, economical, rapid, and flexible technique allowing sensitive parallel processing of many samples on one plate [39]. Therefore, the TLC is widely used for the qualitative analysis of naphthoquinones in plant raw materials, substances, and phytodrugs. When analyzing extracts in the mobile phase hexane-acetone (20 : 1), red spots were observed, the colours of which changed to violet on treatment with ammonia vapour. Based on the colour, the secondary metabolites were differentiated and Rf values were calculated. The separation of extracts with TLC is presented in Figure 2.

A comparative chromatographic evaluation showed the presence of two shikonin derivatives in all the tested extracts of the roots of *O. gmelinii*. The spots with an Rf value of approximately 0.2 were shikonin [40].

For the quantitative analysis of shikonin and its derivatives spectrophotometric method was used, since most naphthoquinones, when dissolved in chloroform, have a maximum light absorption at a wavelength of 525 nm. The amount of naphthoquinones was calculated in terms of shikonin by specific absorption rate shikonin (*A*_1*см*_^1%^=430). The results are presented in Table 2.

The concentration of naphthoquinone in extracts was significantly affected by the method of obtaining the extract, the amount of plant raw materials, and the % yield of extract. The amount of naphthoquinones in terms of shikonin in the CO_2_ extract exceeds the amount of naphthoquinones in the extracts performed by ultrasonic extraction and percolation by 13 and 30 times, respectively.

Studies conducted in recent years by various authors have shown that shikonin and its derivatives possess many diverse properties, including antioxidant [41, 42], anti-inflammatory [43], antiviral [44–46], antifungal [47], antiparasitic [48], antibacterial [49], wound healing [50], and even anticancer effects [51–53].

At the same time, other phytochemical components found in extracts also have biological and therapeutic activities. In particular, squalene is triterpene, which acts as an antibacterial, antioxidant, antitumor, immunostimulant, chemopreparative, lipoxygenase inhibitor, and anti-HIV [54]. Hexadecanoic acid (palmitic acid) has anti-inflammatory, antioxidant, hypocholesterolemic, and antibacterial activity [55]. Oleic acid has antibacterial potential [56]. Linoleic acid (9,12-octadecadienoic acid) and linolenic acid (9,12,15-octadecatrienoic acid) represent two families of essential polyunsaturated fatty acids (Omega-6 and Omega-3). They are the precursors of their higher molecular weight and more unsaturated components. The ultimate metabolites of PUFA are various prostaglandins. A lack of PUFA in the human body can lead to the development of a broad spectrum of diseases such as cardiovascular pathologies, inflammatory processes, viral infections, autoimmune diseases, and certain types of cancer [57].

Summing up the results of the component composition, we decided to study the biological properties of the extracts, in particular antiviral and antimicrobial activity.

### 3.2. Biological Activity of Extracts

#### 3.2.1. Antiviral Activity

The various root extracts of *Onosma gmelinii* were tested for cytotoxicity and antiviral activity. To assess the antiviral activity of plant extracts in experiments *in vitro*, their nontoxic concentrations were used. The viability of MDCK cells incubated in the presence or absence of the extracts was evaluated by MTT assay. The CC_50_ values of extracts of *Onosma gmelinii* obtained by supercritical CO_2_ extraction, ultrasound-assisted extraction, and percolation extraction were 7.5, 200.0, and 236.6 *μ*g/mL (*p* < 0.05), respectively.

Evaluation of antiviral activity of plant extracts against different strains of influenza viruses A (A/FPV/Weybridge/78 (H7N7), A/Swine/Iowa/15/30 (H1N1), A/black-headed gull/Atyrau/743/04 (H13N6), A/FPV/Rostock/1934 (H7N1), A/Almaty/8/98 (H3N2)) was carried out using therapeutic and viral inhibition schemes. For all extracts, IC_50_ and SI were determined. The SI value most reliably characterizes the specific antiviral activity of the compound under study. The higher the SI value is, the more effective the compound is considered to be against the virus. The results are presented in Tables 3 and 4.

It was revealed that the highest therapeutic activity is shown by CO_2_ extract against influenza strains A/H7N7, A/H13N6, and A/H7N1 with IC_50_ value of 1.2, 1.2, and 0.9 *µ*g/mL (*p* < 0.05), respectively. At the same time, the selectivity index (SI) was 6.2, 6.2, and 8.3, respectively. The ultrasonic extract is active against influenza strains A/H7N7, A/H1N1, A/H7N1, and A/H3N2, with SI values ranging from 4 to 5. It is possible to hypothesize that the CO_2_ extract and ultrasonic extract can pass through cell membranes and target the viral cycle's intracellular stages, such as replication, particle assembly, or budding. The percolation extraction showed insignificant activity against influenza viruses A; the SI value did not exceed 3.

As shown in Table 4 the percolation extraction had the highest antiviral activity against influenza virus strain A/H1N1 with SI = 93.5, followed by the ultrasound-assisted extraction (SI = 38.0) and supercritical CO_2_ extraction (SI = 11.7). However, the ultrasonic extract displayed the highest antiviral activity against the influenza virus strain A/H7N7 (IC_50_ = 20.2 *µ*g/mL (*p* < 0.05), SI = 9.9). It is possible to assume that these extracts potentially exert antiviral activity via blockage of viral attachment to sialic acids at the host cell surface. Inhibition of virus attachment, in turn, prevents virus entry, replication, and occurrence of infection. For other influenza virus subtypes, these extracts either are ineffective or show insignificant inhibitory activity with SI = 3.

A total of 190 extracts of 95 medicinal plants traditionally used in Lingnan Chinese Medicines, including those from the roots of *O. gmelinii*, were screened for their neuraminidase (NA) inhibitory activities for influenza virus A/H1N1 by Liu et al. The EtOAc (ethyl acetate) and MeOH (methanol) extracts from the roots of *O. gmelinii* at a final concentration of 40.0 *μ*g/mL were able to inhibit NA of influenza virus A/H1N1 by 40% and 15.9%, respectively [30].

#### 3.2.2. Antimicrobial Activity

Antibacterial and antifungal activities of *O. gmelinii* extracts were tested against a panel of microbes: two Gram-negative bacteria (*E. coli* ATCC 8739 and *Pseudomonas aeruginosa* ATCC 9027); nine Gram-positive bacteria (*Staphylococcus aureus* ATCC 6538-P, *Staphylococcus aureus* ATCC BAA-39 (multidrug resistant, MRSA), *Staphylococcus epidermidis* ATCC 51625 (methicillin resistant), *Staphylococcus epidermidis* ATCC 12228 (VRSA), *Streptococcus pyogenes* ATCC 19615, *Streptococcus pneumoniae* ATCC BAA-660 (multidrug resistant), *Enterococcus hirae* ATCC 10541, *Enterococcus faecalis* ATCC 51575 (gentamicin, streptomycin, and vancomycin resistant), and *Enterococcus faecium* ATCC 700221 (vancomycin and teicoplanin resistant)), and two fungal species (*Candida albicans* ATCC 10231 and *Candida albicans* ATCC 2091). Ampicillin is a broad-spectrum beta-lactam antibiotic active against Gram-positive and Gram-negative bacteria and nystatin is an antifungal medicine highly active against yeast-like fungi of the genus *Candida* and they were used as reference drugs. The results of the research of antimicrobial activity by serial dilution are reported in Table 5.

The extracts were inactive against Gram-negative bacteria such as *Escherichia coli*, *Pseudomonas aeruginosa*. In contrast, all Gram-positive bacteria were sensitive to various extracts of root plant. Percolation extraction has the strongest effect on staphylococci: *Staphylococcus aureus* ATCC 6538-P, *Staphylococcus aureus* ATCC BAA-39, *Staphylococcus epidermidis* ATCC 51625, and *Staphylococcus epidermidis* ATCC 12228 (MBC of 4687.5 *µ*g/mL, 4687.5 *µ*g/mL, 2343.8 *µ*g/mL, 4687.5 *µ*g/mL, respectively). Ultrasonic extraction is active against a panel of Gram-positive bacteria such as *Staphylococcus aureus*, *Staphylococcus epidermidis*, *Streptococcus pneumoniae*, *Enterococcus hirae*, and *Enterococcus faecium* at MBCs ranging from 18.3 to 293.0 *µ*g/mL. The greatest bactericidal activity against Gram-positive bacteria was shown by the CO_2_ extract of *Onosma gmelinii*. It turned out to be 100–200 times more effective than reference drug (ampicillin).

Only CO_2_ extract and ultrasonic extract were effective against fungi, inhibiting the growth of *Candida albicans* ATCC 10231 at concentrations of 48.8 and 1171.9 *µ*g/mL, respectively, and *Candida albicans* ATCC 2091 at concentrations 97.7 and 2343.8 *µ*g/mL, respectively.

The reason for higher sensitivity of Gram-positive bacteria in comparison with negative bacteria could be explained by differences between their cell wall compositions. The cell wall of Gram-positive bacteria contains a thick homogeneous peptidoglycone layer, which is an ineffective permeability barrier, while the high resistance of Gram-negative bacteria to external agents can be attributed to the presence of an outer membrane formed by proteins, phospholipids and lipopolysaccharides, which acts as a barrier protecting the cell from penetration of many compounds (antibiotics, detergents, hydrophilic dyes, etc.).

The results of our study are well consistent with the findings of Moghaddam et al. [58], who reported that the root extracts of O*nosma dichroanthum* had antimicrobial efficiency against Gram-positive bacteria, but no effect on Gram-negative bacteria. Ozgen et al. [59] proved that the root extract of *Onosma argentatum* has antioxidant and antimicrobial effects agent against *S. aureus*, *Bacillus subtilis*, and *E. coli* but is inactive against *Candida albicans*.

A lot of research is focused on studying the antimicrobial activity not so much of crude extracts from various parts of plants of the family Boraginaceae, but natural products isolated from them. So Shen et al. [49] showed the inhibitory activity of shikonin and some of its derivatives against methicillin-resistant *S. aureus* and vancomycin-resistant *E. faecium* and *E. faecalis*. Miao et al. [47] demonstrated that shikonin is active against C. albicans, including several fluconazole-resistant strains, against which shikonin (MIC_80_ value 4.0 *µ*g/mL) was shown to be >16 times more potent than the standard (MIC_80_ > 64.0 *µ*g/mL).

## 4. Conclusion

For the first time, the chemical composition and biological activity of various extracts of the *Onosma gmelinii* roots growing in the territory of the Republic of Kazakhstan were determined. The main biologically active substances of raw materials are naphthoquinone derivatives. The preparation method has a great influence on the concentration of naphthoquinones in the extract. So the amount of naphthoquinones after supercritical carbon dioxide extraction was about 40%, after ultrasound-assisted extraction about 3%, and after percolation extraction about 1.3%. GC-MS analysis showed that ultrasonic extraction makes it possible to isolate a larger number of phytochemical components compared to CO_2_ extraction and percolation extraction. In addition to triterpenes, monocarboxylic acids, fatty acids, and their derivatives, including essential polyunsaturated fatty acids identified in all obtained extracts, flavonoids, and 5-Hydroxymethylfurfural, which have high biological activity, were identified in the ultrasonic extract.

The results of the antiviral action showed that, of all the studied extracts, the ultrasonic extract has the greatest effectiveness against different subtypes of the influenza virus A, showing both therapeutic and virus inhibitory activity. CO_2_ extract showed high therapeutic activity, but it was not effective for direct exposure to viral particles.

Study of antimicrobial activity showed that all tested extracts are effective against Gram-positive bacteria but are inactive against Gram-negative bacteria. Percolation extract shows low efficacy against Gram-positive bacteria (MBC from 2343.8 to 4687.5 *µ*g/mL), compared to the reference drug (MBC from 3.9 to 500.0 *µ*g/mL). Ultrasonic extract of *Onosma gmelinii* has a significant effect against a panel of Gram-positive bacteria (*Staphylococcus aureus* ATCC 6538-P, *Staphylococcus aureus* ATCC BAA-39, *Staphylococcus epidermidis* ATCC 51625, *Staphylococcus epidermidis* ATCC 12228, *Streptococcus pneumoniae* ATCC BAA-660, *Enterococcus hirae* ATCC 10541, and *Enterococcus faecium* ATCC 700221), with MBC from 18.3 to 293.0 *µ*g/mL. CO_2_ extract of *Onosma gmelinii* has the greatest bactericidal activity against Gram-positive bacteria; it is 100–200 times more effective than ampicillin. Antifungal activity is observed only for two extracts: CO_2_ extract and ultrasonic extract.

The obtained results indicate that ultrasonic extraction is the optimal method for obtaining an extract from the roots of *Onosma gmelinii*. The extract has antiviral and antimicrobial activity with a cheap preparation method. At the same time, it is also worth noting that the CO_2_ extract has a high antibacterial effect. These extracts can be further used for the development of phytopreparations for medical practice.

## Figures and Tables

**Table 1:** GC-MS analysis of *O. gmelinii* root extracts.

No.	Compound name	Molecular formula	СО_2_ extract		Ultrasonic extract		Percolation extract	
			Identification probability (%)	Percentage (%)	Identification probability (%)	Percentage (%)	Identification probability (%)	Percentage (%)
1	Acetic acid	C2H4O2	97	15.53	97	10.83	97	10.87
2	Propanoic acid	C3H6O2	95	0.5	91	0.19	84	0.12
3	Propanoic acid, 2-methyl-	C4H8O2	93	13.99	93	5.69	94	4.45
4	2-Butenoic acid, 3-methyl-	C5H8O2	87	10.11	95	9.24	96	9.97
5	3-Methyl-3-butenoic acid	C5H8O2	88	1.63	89	0.71	88	0.6
6	2-Butenoic acid, 2-methyl-	C5H8O2	95	0.6	93	0.25	81	0.17
7	Butanoic acid, 3-hydroxy-3-methyl-	C5H10O3	74	6.34	74	1.89	72	0.37
8	2-Methoxy-4-vinylphenol	C9H10O2	90	0.15	92	1.32	92	1.05
9	Hexadecanoic acid, ethyl ester	C18H36O2	90	0.64	86	0.28	91	6.52
10	9,12-Octadecadienoic acid, ethyl ester	C20H36O2	88	0.7	84	0.56	92	4.05
11	Hexadecanoic acid	C16H32O2	93	3.84	93	2.14	92	1.92
12	Squalene	C30H50	97	2.63	78	0.72	88	0.56
13	Oleic Acid	C18H34O2	95	4.37	89	1.65	82	0.5
14	9,12-Octadecadienoic acid	C18H32O2	91	7.0	92	2.43	91	1.53
15	9,12,15-Octadecatrienoic acid	C18H30O2	91	2.17	78	1.23	73	0.98
16	Nonanoic acid	C9H18O2	90	0.61	77	0.15	—	—
17	4-Methylphthalaldehyde	C9H8O2	74	0.18	71	0.16	—	—
18	9,12,15-Octadecatrienoic acid, methyl ester	C19H32O2	84	0.16	78	0.18	—	—
19	Palmitoleic acid	C16H30O2	94	1.34	76	0.34	—	—
20	Octadecanoic acid	C17H35CO2H	91	1.03	83	0.51	—	—
21	n-Tetracosanol-1	C24H50O	94	2.88	86	1.65	—	—
22	Ethyl 9-hexadecenoate	C18H34O2	87	0.24	—	—	91	0.77
23	Octadecanoic acid, ethyl ester	C20H40O2	69	0.11	—	—	90	0.79
24	Ethyl oleate	C20H38O2	89	0.37	—	—	90	3.1
25	Methyl stearidonate	C19H30O2	80	0.06	—	—	86	0.41
26	2,2′-Bioxirane	C4H6O2	—	—	83	0.59	81	0.48
27	2-Propanone, 1-(acetyloxy)-	C5H8O3	—	—	75	0.22	81	0.19
28	Formic acid	CH2O2	—	—	96	0.48	85	0.27
29	2,4-Dihydroxy-2,5-dimethyl-3(2H)-furan-3-one	C6H8O4	—	—	88	0.35	87	0.28
30	4-Cyclopentene-1,3-dione	C5H4O2	—	—	86	0.35	88	0.57
31	Butanoic acid, 4-hydroxy-	C4H8O3	—	—	91	0.18	90	0.24
32	Carbamic acid, methyl ester	C2H5NO2	—	—	93	0.16	90	0.27
33	2-Furanmethanol	C5H6O2	—	—	96	1.29	95	1.19
34	Hexanoic acid, 2-methyl-	C7H14O2	—	—	81	10.29	81	10.67
35	1,2-Cyclopentanedione	C5H6O2	—	—	93	0.26	92	0.49
36	Urea, 1-methylcyclopropyl-	C5H10N2O	—	—	72	0.41	72	1.53
37	2,5-Dimethyl-4-hydroxy-3(2H)-furanone	C6H8O3	—	—	85	1.04	84	1.07
38	2H-Pyran-2,6(3H)-dione	C5H4O3	—	—	81	0.15	86	0.25
39	Dihydroxyacetone	C3H6O3	—	—	78	1.64	80	1.2
40	Cyclopropyl carbinol	C4H8O	—	—	74	0.6	78	0.93
41	1,3-Dioxol-2-one, 4,5-dimethyl-	C5H6O3	—	—	77	0.87	77	0.7
42	2-Hydroxy-gamma-butyrolactone	C4H6O3	—	—	78	0.98	85	1.65
43	4H-Pyran-4-one, 2,3-dihydro-3,5-dihydroxy-6-methyl-	C6H8O4	—	—	90	3.51	91	4.52
44	Glycerin	C3H8O3	—	—	92	2.09	86	0.87
45	2-Propanamine, N-methyl-N-nitroso-	C4H10N2O	—	—	70	0.65	72	0.89
46	Benzofuran, 2,3-dihydro-	C8H8O	—	—	80	0.17	84	0.21
47	5-Hydroxymethylfurfural	C6H6O3	—	—	92	10.67	91	8.56
48	2(3H)-Furanone, dihydro-4-hydroxy-	C4H6O3	—	—	78	0.22	77	0.22
49	Sucrose	C12H22O11	—	—	74	4.01	74	5.71
50	Nonane, 1,1-diethoxy-	C13H28O2	88	0.38	—	—	—	—
51	Butanoic acid, 3-methyl-	C5H10O2	86	16.26	—	—	—	—
52	Hexadecane, 2,6,11,15-tetramethyl-	C20H42	82	0.28	—	—	—	—
53	Heptanoic acid	C7H14O2	69	0.09	—	—	—	—
54	Heptadecane, 2,6,10,15-tetramethyl-	C21H44	76	0.07	—	—	—	—
55	Octanoic acid	С8Н16О2	83	0.06	—	—	—	—
56	2-Pentadecanone, 6,10,14-trimethyl-	C18H36O	76	0.10	—	—	—	—
57	Hentriacontane	C31H64	71	0.25	—	—	—	—
58	Dodecanoic acid	C12H24O2	78	0.10	—	—	—	—
59	9,12-Octadecadienoyl chloride	C18H31ClO	83	0.23	—	—	—	—
60	1-Hexadecanol	C16H34O	85	0.22	—	—	-—	—
61	Ethyl 9,12,15-octadecatrienoate	C20H34O2	87	0.19	—	—	—	—
62	Methyl 19-methyl-eicosanoate	C22H44O2	71	0.08	—	—	—	—
63	1,2-Benzenedicarboxylic acid, butyl 8-methylnonyl ester	C22H34O4	75	0.26	—	—	—	—
64	1-Docosene	C22H44	83	0.64	—	—	—	—
65	17-Pentatriacontene	C35H70	71	0.57	—	—	—	—
66	Docosanoic acid, ethyl ester	C24H48O2	70	0.14	—	—	—	—
67	Behenic alcohol	C22H46O	91	1.22	—	—	—	—
68	Ethyl tetracosanoate	C26H52O2	81	0.44	—	—	—	—
69	Ethyl (2E)-3-(4-hydroxy-3-methoxyphenyl)-2-propenoate	C12H14O4	74	0.18	—	—	—	—
70	Gamolenic acid	C18H30O2	86	1.07	—	—	—	—
71	Hydrazoic acid	HN3	—	—	74	0.03	—	—
72	2,3-Butanediol	C4H10O2	—	—	92	0.12	—	—
73	2(5H)-Furanone, 5-methyl-	C5H6O2	—	—	67	0.15	—	—
74	Furfural	C5H4O2	—	——	76	2.39	—	—
75	2-Furancarboxaldehyde, 5-methyl-	C6H6O2	—	—	87	0.15	—	—
76	2(5H)-Furanone	C4H4O2	—	—	94	0.10	—	—
77	Butanoic acid, 2-ethyl-, methyl ester	C7H14O2	—	—	63	0.32	—	—
78	3-Furancarboxylic acid, methyl ester	C6H6O3	—	—	83	0.13	—	—
79	5-Acetoxymethyl-2-furaldehyde	C8H8O4	—	—	74	0.15	—	—
80	4H-Pyran-4-one, 3,5-dihydroxy-2-methyl-	C6H6O4	—	—	71	0.14	—	—
81	5-Hydroxymethyldihydrofuran-2-one	C5H8O3	—	—	81	0.17	—	—
82	1,2-Ethanediol, 1-(2-furanyl)-	C6H8O3	—	—	64	0.43	—	—
83	DL-Proline, 5-oxo-, methyl ester	C6H9NO3	—	—	76	0.24	—	—
84	1,2-Benzenedicarboxylic acid, butyl octyl ester	C20H30O4	—	—	63	0.19	—	—
85	Glyceraldehyde	C3H6O3	—	—	68	0.23	—	-
86	Uric acid	C5H4N4O3	—	—	64	0.21	—	—
87	Pentadecanoic acid	C15H30O2	—	—	68	0.17	—	—
88	2-Acetylthiazole	C5H5NOS	—	—	61	0.16	—	—
89	Hexadecenoic acid, Z-11-	C16H30O2	—	—	91	0.75	—	—
90	1-Nonadecene	C19H38	—	—	73	0.28	—	—
91	9,10-Anthracenedione, 1,8-dihydroxy-3-methyl	C15H10O4	—	—	74	3.42	—	—
92	Bis(2-ethylhexyl) phthalate	C24H38O4	—	—	95	6.14	—	—
93	1-Eicosanol	C20H42O	—	—	77	0.42	—	—
94	D-Allose	C6H12O6	—	—	69	0.25	—	—
95	Acetic acid, (acetyloxy)-	C4H6O4	—	—	—	—	75	0.15
96	Acetic acid, hydroxy-, methyl ester	C3H6O3	—	—	—	—	89	0.36
97	Propanoic acid, 2-oxo-, methyl ester	C4H6O3	—	—	—	—	77	2.86
98	2-Furanmethanol, 5-methyl-	C6H8O2	—	—	—	—	77	0.13
99	*γ*-Linolenic acid, methyl ester	C19H32O2	—	—	—	—	83	0.71
100	9,12,15-Octadecatrienoic acid, ethyl ester	C20H34O2	—	—	—	—	93	1.68
101	15-Tetracosenoic acid, methyl ester	C25H48O2	—	—	—	—	70	0.37
102	Diisooctyl phthalate	C24H38O4	—	—	—	—	88	2.06
Total compounds for each solvent		—	46		69		51	

**Table 2:** Content of the sum of naphthoquinones in extracts from *O. gmelinii*.

Extract name	Amount of naphthoquinones (% ± SD)
Percolation (*P*)	1.30 ± 0.02
Ultrasound-assisted extraction (UAE)	2.98 ± 0.04
Supercritical carbon dioxide extraction (CO2-SE)	39.57 ± 0.55

**Table 3:** Therapeutic activity of *O. gmelinii* root extracts on influenza viruses A.

No.	Influenza virus strains	^a^СO2-SE			^b^UAE			^c^P		
		^d^CC_50_, *µ*·g/mL	^e^IC_50_, *µ*·g/mL	^f^SI	CC_50_, *µ*·g/mL	IC_50_, *µ*·g/mL	SI	CC_50_, *µ*·g/mL	IC_50_, *µ*·g/mL	SI
1	А/FPV/Weybridge/78 (H7N7)	7.5	1.2	6.2	200.0	44.9	4.5	236.6	76.9	3.1
2	A/Swine/Iowa/15/30 (H1N1)		3.6	2.1		49.0	4.1		80.6	2.9
3	A/black-headed gull/Atyrau/743/04 (H13N6)		1.2	6.2		71.8	2.8		88.7	2.7
4	A/FPV/Rostock/1934 (H7N1)		0.9	8.3		41.8	4.8		82.8	2.9
5	А/Almaty/8/98 (H3N2)		3.2	2.4		42.6	4.7		99.7	2.4

^a^CO2-SE: supercritical carbon dioxide extraction; ^b^UAE: ultrasound-assisted extraction; ^c^P: percolation extraction; ^d^CC_50_: median (50%) cytotoxic concentration in MDCK cells; ^e^IC_50_: inhibitory concentration required to protect 50% of cells; and ^f^SI: selectivity index, CC_50_/IC_50_.

**Table 4:** Virus inhibition activity of *O. gmelinii* root extracts on influenza viruses A.

No.	Influenza virus strains	^a^СO2-SE			^b^UAE			^c^P		
		^d^CC_50_, *µ*·g/mL	^e^IC_50_, *µ*·g/mL	^f^SI	CC_50_, *µ*·g/mL	IC_50_, *µ*·g/mL	SI	CC_50_, *µ*·g/mL	IC_50_, *µ*·g/mL	SI
1	А/FPV/Weybridge/78 (H7N7)		^g^ND	ND		20.2	9.9		80.0	3.0
2	A/Swine/Iowa/15/30 (H1N1)		0.6	11.7		5.3	38.0		2.5	93.5
3	A/black-headed gull/Atyrau/743/04 (H13N6)	7.5	ND	ND	200	70.6	2.8	236.6	87.2	2.7
4	A/FPV/Rostock/1934 (H7N1)		ND	ND		ND	ND		ND	ND
5	А/Almaty/8/98 (H3N2)		ND	ND		0.0654	3.1		77.4	3.1

^a^CO2-SE: supercritical carbon dioxide extraction; ^b^UAE: ultrasound-assisted extraction; ^c^P: percolation extraction; ^d^CC_50_: median (50%) cytotoxic concentration in MDCK cells; ^e^IC_50_: inhibitory concentration required to protect 50% of cells; ^f^SI: selectivity index, CC_50_/IC_50_; and ^g^ND: not determined.

**Table 5:** The antimicrobial activity results of *O. gmelinii* extracts obtained by the method of serial dilutions.

No.	Strain name	^a^СO2-SE	^b^UAE	^c^P	^d^reference drugs
		^e^MBC or ^f^MFC (*μ*·g/ml)			
*Gram-negative bacteria*					

1	*Escherichia coli* ATCC 8739	12500.0	^g^ND	ND	62.5
2	*Pseudomonas aeruginosa* ATCC 9027	12500.0	150000.0	150000.0	31.3

*Gram-positive bacteria*					

3	*Staphylococcus aureus* ATCC 6538-P	0.4	73.3	4687.5	3.9
4	*Staphylococcus aureus* ATCC BAA-39	0.8	73.3	4687.5	62.5
5	*Staphylococcus epidermidis* ATCC 51625	0.1	18.3	2343.8	7.8
6	*Staphylococcus epidermidis* ATCC 12228	0.4	73.3	4687.5	31.3
7	*Streptococcus pyogenes* ATCC 19615	390.6	9375.0	9375.0	7.8
8	*Streptococcus pneumoniae* ATCC BAA-660	24.4	293.0	2343.8	0.02
9	*Enterococcus hirae* ATCC 10541	6.1	146.5	ND	62.5
10	*Enterococcus faecalis* ATCC 51575	6.1	2343.8	ND	1000.0
11	*Enterococcus faecium* ATCC 700221	3.1	73.3	2343.8	500.0

*Fungi*					

12	*Candida albicans* ATCC 10231	48.8	1171.9	ND	2.7
13	*Candida albicans* ATCC 2091	97.7	2343.8	ND	0.2

^a^CO2-SE: supercritical carbon dioxide extraction; ^b^UAE: ultrasound-assisted extraction; ^c^P: percolation extraction; ^d^reference drugs: ampicillin for bacteria and nystatin for fungi; ^e^MBC: minimum bactericidal concentration; ^f^MFC: minimum fungicidal concentration; and ^g^ND: not determined.

## Data Availability

The data used to support the findings of this study are available from the corresponding author on reasonable request.

## References

[1] Katanic Stankovic J. S., Ceylan R., Zengin G. (2020). Multiple biological activities of two *Onosma* species (*O. sericea* and *O. stenoloba*) and HPLC-MS/MS characterization of their phytochemical composition. *Industrial Crops and Products*.

[2] Cragg G. M., Newman D. J. (2013). Natural products: a continuing source of novel drug leads. *Biochimica et Biophysica Acta (BBA)-General Subjects*.

[3] WHO (2003). *WHO Guidelines on Good Agricultural and Collection Practices (GACP) for Medicinal Plants*.

[4] WHO (2012). *Bulletin of the World Health Organization: Special Collection [Russian]*.

[5] Kumar N., Kumar R., Kishore K. (2013). *Onosma* L.: a review of phytochemistry and ethnopharmacology. *Pharmacognosy Reviews*.

[6] Sarikurkcu C., Sahinler S. S., Husunet M. T., Istifli E. S., Tepe B. (2020). Two endemic *Onosma* species (*O. sieheana* and *O. stenoloba*): a comparative study including docking data on biological activity and phenolic composition. *Industrial Crops and Products*.

[7] Jabbar A. A. j (2021). *Onosma* mutabilis: phytochemical composition, antioxidant, cytotoxicity, and acute oral toxicity. *Food Sciences and Nutrition*.

[8] Azarfar A., Rafiee Z., Ravanshad Y., Saber Moghadam N., Bakhtiari E. (2020). Effect of herbal formulation “Cystone®” on urolithiasis. *Jundishapur Journal of Natural Pharmaceutical Products*.

[9] Hu Y., Jiang Z., Leung K. S.-Y., Zhao Z. (2006). Simultaneous determination of naphthoquinone derivatives in Boraginaceous herbs by high-performance liquid chromatography. *Analytica Chimica Acta*.

[10] Ozgen U., Ikbal M., Hacimuftuoglu A. (2006). Fibroblast growth stimulation by extracts and compounds of *Onosma* argentatum roots. *Journal of Ethnopharmacology*.

[11] Roeder E., Wiedenfeld H. (2009). Pyrrolizidine alkaloids in medicinal plants of Mongolia, Nepal and Tibet. *Die Pharmazie*.

[12] Ahmad I., Nawaz S. A., Afza N. (2005). Isolation of onosmins A and B, lipoxygenase inhibitors from *Onosma* hispida. *Chemical and Pharmaceutical Bulletin*.

[13] Gruzinskaya L. M., Gemejiyeva N. G., Nelina N. V., Karzhaubekova Z. (2014). *An Annotated List of Medicinal Plants of Kazakhstan*.

[14] Naz S., Ahmad S., Ajaz Rasool S., Asad Sayeed S., Siddiqi R. (2006). Antibacterial activity directed isolation of compounds from *Onosma* hispidum. *Microbiological Research*.

[15] Ahmad B., Ali N., Bashir S., Choudhary M. I., Azam S., Khan I. (2009). Parasiticidal, antifungal and antibacterial activities of *Onosma griffithii* Vatke. *African Journal of Biotechnology*.

[16] Sarikurkcu C., Sahinler S. S., Tepe B. (2020). *Onosma aucheriana*, *O. frutescens*, and *O. sericea*: phytochemical profiling and biological activity. *Industrial Crops and Products*.

[17] Pal M., Chaudhury A. (2010). High frequency direct plant regeneration, micropropagation and Shikonin induction in Arnebia hispidissima. *Journal of Crop Science and Biotechnology*.

[18] Noula E., Samanidou V. F., Assimopoulou A. N., Papageorgiou V. P., Papadoyannis I. N. (2010). Solid-phase extraction for purification of alkannin/shikonin samples and isolation of monomeric and dimeric fractions. *Analytical and Bioanalytical Chemistry*.

[19] Andújar I., Ríos J. L., Giner R. M., Recio M. D. (2013). Pharmacological properties of shikonin–a review of literature since 2002. *Planta Medica*.

[20] https://www.efloras.org/florataxon.aspx?flora_id=2&taxon_id=200019154.

[21] Ishmuratova M. Yu. Useful plants of the Karaganda region.

[22] Azwanida N. N. (2015). A Review on the extraction methods use in medicinal plants, principle, strength and limitation. *Medicinal & Aromatic Plants*.

[23] Papageorgiou V. P., Assimopoulou A. N., Samanidou V. F., Papadoyannis I. N. (2006). Analytical methods for the determination of alkannins and shikonins. *Current Organic Chemistry*.

[24] Eruygur N. (2018). A simple isocratic high-perfomance liquid chromatography method for the simultaneous determination of shikonin derivatives in some Echium species growing wild in Turkey. *Turkish Journal of Pharmaceutical Sciences*.

[25] Amirkhanova A., Ustenova G., Krauze M., Poblocka L., Shynykul Z. (2018). Thin-layer chromatography analysis of extract oxytropis glabra LAM. DC. *International Multidisciplinary Scientific GeoConference: Surveying Geology and Mining Ecology Management SGEM*.

[26] Daironas J. V., Zilfikarov I. N., Sokolskaya T. A. (2014). Quantative determination of shikonin and its derivataves in raw material and drugs. *Problems of Biological, Medical and Pharmaceutical Chemistry*.

[27] Szretter K. J., Balish A. L., Katz J. M. (2006). Influenza: propagation, quantification, and storage. *Current Protocols in Microbiology*.

[28] Reed L., Muench H. (1938). A simple method of estimating fifty percent endpoints. *American Journal of Epidemiology*.

[29] Mosmann T. (1983). Rapid colorimetric assay for cellular growth and survival: application to proliferation and cytotoxicity assays. *Journal of Immunological Methods*.

[30] Liu J., Zu M., Chen K. (2018). Screening of neuraminidase inhibitory activities of some medicinal plants traditionally used in Lingnan Chinese medicines. *BMC Complementary and Alternative Medicine*.

[31] Zubenko N., Bekesheva K., Korotetskiy I., Toxanbayev R., Ustenova G. (2017). Study on antiviral activity of coordination compound based on molecular iodine against influenza a virus. *International Multidisciplinary Scientific GeoConference-SGEM*.

[32] Joshi B., Panda S. K., Jouneghani R. S. (2020). Antibacterial, antifungal, antiviral, and anthelmintic activities of medicinal plants of Nepal selected based on ethnobotanical evidence. *Evidence-Based Complementary and Alternative Medicine*.

[33] Liu A.-L., Liu B., Qin H.-L., Lee S., Wang Y.-T., Du G.-H. (2008). Anti-Influenza virus activities of flavonoids from the medicinal plant Elsholtzia rugulosa. *Planta Medica*.

[34] WFCC executive board (2010). *World Federation for Culture Collection Guidelines for the Establishment and Operation of Culture Collections of Cultures of Microorganisms*.

[35] CLSI (2020). *Performance Standards for Antimicrobial Susceptibility Testing*.

[36] CLSI (2017). *Reference Method for Broth Dilution Antifungal Susceptibility Testing of Yeasts*.

[37] Neeraj, Vasudeva N., Sharma S. (2019). Chemical composition of Fagopyrum esceulentum Moench seed through GC-MS. *IJPSR*.

[38] Ababutain I. M., Alghamdi A. I. (2021). *In Vitro* anticandidal activity and gas chromatography-mass spectrometry (GC-MS) screening of *Vitex agnus-castus* leaf extracts. *PeerJ*.

[39] Damianakos H., Kretschmer N., Sykłowska-Baranek K., Pietrosiuk A., Bauer R., Chinou I. (2012). Antimicrobial and cytotoxic isohexenylnaphthazarins from Arnebia euchroma (Royle) Jonst. (Boraginaceae) callus and cell suspension culture. *Molecules*.

[40] Dayronas J. V., Zilfikarov I. N. (2011). *Natural Naphthoquinones: Prospects for Medical Use*.

[41] Jin R., Bai Y. (2012). Theoretical investigation of the radical scavenging activity of shikonin and acylshikonin derivatives. *Journal of Molecular Modeling*.

[42] Nishizawa M., Kohno M., Nishimura M., Kitagawa A., Niwano Y. (2005). Presence of peroxyradicals in cigarette smoke and the scavenging effect of shikonin, a naphthoquinone pigment. *Chemical and Pharmaceutical Bulletin*.

[43] Lu L., Qin A., Huang H. (2011). Shikonin extracted from medicinal Chinese herbs exerts anti-inflammatory effect via proteasome inhibition. *European Journal of Pharmacology*.

[44] Li H. M., Tang Y. L., Zhang Z. H. (2012). Compounds from Arnebia euchroma and their related anti-HCV and antibacterial activities. *Planta Medica*.

[45] Chen X., Yang L., Zhang N. (2003). Shikonin, a component of Chinese herbal medicine, inhibits chemokine receptor function and suppresses human immunodeficiency virus type 1. *Antimicrobial Agents and Chemotherapy*.

[46] Gao H., Liu L., Qu Z. Y. (2011). Anti-adenovirus activities of shikonin, a component of Chinese herbal medicine in vitro. *Biological and Pharmaceutical Bulletin*.

[47] Miao H., Zhao L., Li C. (2012). Inhibitory effect of shikonin on Candida albicans growth. *Biological and Pharmaceutical Bulletin*.

[48] Ali A., Assimopoulou A. N., Papageorgiou V. P., Kolodziej H. (2011). Structure/antileishmanial activity relationship study of naphthoquinones and dependency of the mode of action on the substitution patterns. *Planta Medica*.

[49] Shen C. C., Syu W. J., Li S. Y., Lin C. H., Lee G. H., Sun C. M. (2002). Antimicrobial activities of naphthazarins from Arnebia euchroma. *Journal of Natural Products*.

[50] Papageorgiou V. P., Assimopoulou A. N., Ballis A. C. (2008). Alkannins and shikonins: a new class of wound healing agents. *Current Medicinal Chemistry*.

[51] Kretschmer N., Rinner B., Deutsch A. J. A. (2012). Naphthoquinones from *Onosma* paniculata induce cell-cycle arrest and apoptosis in melanoma Cells. *Journal of Natural Products*.

[52] Chen J., Xie J., Jiang Z., Wang B., Wang Y., Hu X. (2011). Shikonin and its analogs inhibit cancer cell glycolysis by targeting tumor pyruvate kinase-M2. *Oncogene*.

[53] Andújar I., Recio M. D., Giner R. M., Ríos J. L. (2013). Traditional Chinese medicine remedy to jury: the pharmacological basis for the use of shikonin as an anticancer therapy. *Current Medicinal Chemistry*.

[54] Amudha M., Rani S. (2014). GC-MS analysis of bioactive components of Cordia retusa (Boraginaceae). *Hygeia: Journal of Drugs and Medicines*.

[55] Abubakar M. N., Majinda R. R. T. (2016). GC-MS analysis and preliminary antimicrobial activity of Albizia adianthifolia (Schumach) and *Pterocarpus angolensis* (DC). *Medicines (Basel)*.

[56] Awa E. P., Ibrahim S., Ameh D. A. (2012). GC/MS analysis and antimicrobial activity of diethyl ether fraction of methanolic extract from the stem bark of *Annona senegalensis* Pers. *International Journal of Pharmaceutical Sciences and Research*.

[57] Yunusova S. G., Khatmulina L. I., Fedorov N. I., Ermolaeva N. A., Galkin E. G., Yunusov M. S. (2012). Polyunsaturated fatty acids from several plant species of the family Boraginaceae. *Chemistry of Natural Compounds*.

[58] Moghaddam P. Z., Mazandarani M., Zolfaghari M. R. (2012). Antibacterial and antioxidant activities of root extract of Onosma dichroanthum Boiss. in north of Iran. *African Journal of Microbiology Research*.

[59] Ozgen U., Houghton P. J., Ogundipe Y., Coşkun M. (2003). Antioxidant and antimicrobial activities of Onosma argentatum and Rubia peregrina. *Fitoterapia*.

